# Recovery of valuable metals from waste printed circuit boards using organic acids synthesised by *Aspergillus niveus*

**DOI:** 10.1049/nbt2.12001

**Published:** 2021-02-07

**Authors:** Santhosh Krishnamoorthy, Gnanasekaran Ramakrishnan, Balaji Dhandapani

**Affiliations:** ^1^ Department of Chemical Engineering Sri Sivasubramaniya Nadar College of Engineering Chennai India; ^2^ Department of Biotechnology Vel Tech High Tech Dr. Rangarajan Dr. Sakunthala Engineering College Chennai India

## Abstract

Organic acids such as citric acid, itaconic acid and oxalic acid synthesised by *Aspergillus niveus* were used for the bioleaching of metals from waste printed circuit boards. Bioleaching of valuable metals was performed in one‐step, two‐steps and spent medium approaches using *A. niveus*. In the absence of waste printed circuit boards (WPCBs), the dry cell weight of *A. niveus* was higher when compared with the presence of WPCBs. Variations in the dry cell weight were observed for the presence of different particle sizes. The increase in itaconic acid and oxalic acid synthesis was found at a reduced particle size (60–80 mesh) and reached the maximum titre of itaconic acid (22.35 ± 0.87 mM) and oxalic acid (12.75 ± 0.54 mM) in 12 days during the two‐step bioleaching. The maximum recovery of 75.66% Zn, 73.58% Ni and 80.25% Cu from WPCBs was achieved in 15 days in two‐step leaching with particle sizes of the mesh being 60–80.

## 1 INTRODUCTION

Modern science with innovative technology generates an enormous amount of electrical and electronic waste materials and it turns out to be a serious environmental issue [1, 2]. The increased production of modern electronic devices has accelerated the generation of an earliest electronic waste beyond measure [3]. Waste printed circuit boards (WPCBs) are one of the main components among the various electrical and electronic equipment's and are rich in several valuable metal contents [4]. Improper disposal of WPCBs threatens the environment because of the harmful toxic compounds present in them [5, 6].

Generally, WPCBs contain precious metals like Cu, Zn, Ni, Al and Fe from 1% to 40 % (wt/wt) on weight basis [7, 8]. Table 1 represents the concentration range of valuable metals from various sources of printed circuit boards [9]. Due to the presence of the expensive components in the WPCBs, various methods were used by many researchers to recycle the WPCBs. To recover the metals from the WPCBs, the two‐step process is used. First, the physical separation results in the removal of physical components and it is followed by the respective extraction process [10]. Methods like pyrolysis [11], gasification [12, 13] pyro metallurgy, co‐combustion, solvent recovery [14] and acid leaching [15, 16] were successfully employed. All these methods had to be performed with utmost care to avoid environmental pollution. Cost is another challenge for recovering metals from WPCBs. Amid these circumstances, an eco‐friendly, cost‐effective method of metal recovery from WPCBs is required.

**TABLE 1:** Composition of metals in WPCBs [9]

Component	Concentration (mg/g)	Component	Concentration (mg/g)
Cu	93.7 – 200.1	Zn	0.32 – 5.3
Ni	2.0 – 11.5	Au	0.002 – 0.003
Fe	2.0 – 52.2	Pb	0.41 – 9.2
Al	10.3 – 35.6	Cr	0.14 – 2.2

Microbial bioleaching method is an eco‐friendly method and could solubilise the metal from the solid state into extractable elements [17, 18, 19]. Metal leaching can be done by two methods namely direct methods and indirect methods. Transformation of protons, oxidation reaction and formation of complex agents are the three basic principles of bioleaching [17]. Bioleaching methodologies are successfully employed for the recovery of the valuable metals like gold, copper and uranium [20, 21]. Heterotrophic bacteria, autotrophic bacterial and fungal species are widely used for the process of bioleaching [22, 23, 24]. The most effective fungi used for bioleaching belong to *Aspergillus* species. These fungal strains produce organic acids and the elimination of protons occurs on the dissociation of organic acid [25]. The roles of citric acid, malic acid oxalic acid and gluconic acid on bioleaching of valuable metals were reported [26, 27]. The impact of itaconic acid on the bioleaching of valuable metals has greater scope for study.

The aim of present investigation is to recover valuable metals like Cu, Zn and Ni from waste WPCBs using bioleaching approaches. First, the collected waste WPCBs were crushed and different sized particles were obtained by sieving. One‐step, two‐steps and spent medium approaches were used for the bioleaching of metals like Zn, Cu and Ni from WPCBs using organic acids synthesised by the isolated *Aspergillus niveus* in the medium containing sucrose. During bioleaching, parameters like dry cell weight, pH, organic acids such as citric acid, itaconic acid and oxalic acid were recorded and the concentration of metals extracted from the WPCBs was analysed.

## 2 MATERIALS AND METHODS

### 2.1 Microorganism and organic acid production

The isolation of the microorganism was performed in the previous study and the identified *A. niveus* was used in the present study [28]. Potato dextrose agar plates were used for the maintenance of *A. niveus* and incubated for 7 days at 30°C. Furthermore, *A. niveus* was cultivated in shake flask cultures (250 ml) containing sterilised culture medium (100 ml) for organic acid production. The production medium contained 120 g/L of sucrose, 1.5 g/L of NaNO_3_, 1.5 g/L of yeast extract, 0.025 g/L of MgSO_4_.7H_2_O and 0.025 g/L of KCl. The recovery of spores was achieved using double distilled water in a sterile condition and about 1 × 10^7^ spores/ml of suspension was prepared using the Haemocytometer. The resultant pore suspension was used as the fungal inoculum.

### 2.2 Preparation of waste printed circuit boards

Waste printed circuits boards of laptops and desktop computers were collected from Vel Tech High Tech Dr. Rangarajan Dr. Sakunthala Engineering College, Tamil Nadu, India. Batteries, plastics and capacitors were physically detached from WPCBs. The collected WPCBs were cut to get small particles [29]. The ball mill was employed to obtain fine particles of the WPCBs and the acquired particles were separated by using a different mesh of 40–60 mesh, 60–80 mesh and <100 mesh according to the method described by Zhu et al. Furthermore, the particles procured were sterilised in autoclave at 121°C for 20 min and stored for further use.

### 2.3 Approaches to bioleaching experiment

The ability of *A. niveus* to bioleach metals from WPCBs was investigated. In order to perform bioleaching experiments in shake flask methods, WPCBs of various particle sizes were added to the organic acid production medium and methods like the one‐step bioleaching, two‐step bioleaching and the spent medium bioleaching were used. Furthermore, bioleaching ability of *A. niveus* was compared with chemical leaching.

#### 2.3.1 One‐step bioleaching

One‐step bioleaching was performed using 0.1% (wt/vol) different sized particles of WPCBs in 250 ml shake flasks containing 100 ml of organic acid production medium. The initial pH was adjusted to 6.0 using 0.1 N NaOH or 0.1 N HNO_3_ solutions. About 1 × 10^7^ of *A. niveus* was inoculated and incubated at 27°C and 120 rpm. The fermentation broth was collected at regular time intervals and filtered to obtain the filtrate. The obtained filtrate was analysed for the estimation of organic acids. The residue, containing both fungal biomass and unleached WPCBs, was heated at two different temperatures (80 and 500°C) and cooled in a desiccator and weighed. The difference in the weight was marked to determine the fungal dry cell weight [17].

#### 2.3.2 Two‐step bioleaching

In two‐step bioleaching 0.1% (wt/vol) of different particle sizes of WPCBs were added to the pre‐culture medium of *A. niveus* after 3 days. Dry cell weight of the fungal biomass and bioleached metal ion concentrations were analysed at various time intervals. At regular time intervals, the amount of organic acids produced by *A. niveus* and dry cell weight of the fungal biomass and the bioleached metal ion concentrations were analysed.

#### 2.3.3 Spent medium leaching

Bioleaching of metals from WPCBs was executed as spent medium approach [30]. Originally, the fermentation broth (100 ml) was collected after 15 days from the organic acid production medium and mycelium of fungi were removed from fermentation broth. About 0.1% (wt/vol) particles of WPCBs were introduced into mycelium free fermentation broth and incubated for 7 days at 27°C under 120 rpm. For every 2 days, samples were collected for the prediction of the metal per cent. Fermentation medium without fungal inoculum was used as control.

#### 2.3.4 Chemical leaching

Chemical leaching experiments were performed in which the different particle sizes of WPCBs were leached individually using strong acids like 100 mM hydrochloric acid and 100 mM sulphuric acid. Later, mixed organic acids consisting of 50 mM citric acid, 30 mM itaconic acid and 20 mM oxalic acid were used for the leaching of WPCBs of different particle size. Analytical grade chemicals were used for chemical leaching. Aliquot samples were collected for the prediction of metal per cent.

### 2.4 Analytical methods

The estimation of organic acids and sugar concentration was performed using high‐performance liquid chromatography (HPLC) (Make: Bio‐Rad, USA). The mobile phase consist of H_2_SO_4_ (0.005 mM) in a column of HPX‐87H. UV‐Vis diode array was employed as the detector at 210 nm. Atomic absorption spectroscopy was used for determining the metal contents like Zn, Cu and Ni. The following Equation (1) was used for metal recovery through bioleaching.

(1) $$\text{Metal}\;\text{recovery}\left(\%\right)=\frac{C_{s}*\;V_{s}}{C_{F}*\;M_{F}}*100\%\;$$

where *C*_S_ is the concentration of metal in leach liquor (mg/L), *V*_S_ is the volume of bioleaching solution (L), *C*_F_ is the amount of metal in the powder (mg/L) and *M*_F_ is the mass of the powder (mg). Scanning Electron Microscope (SEM) (S‐4160, Hitachi, Japan) was used to examine the surface morphology of solid samples. Statistical analysis was performed using Student's *t*‐test using statistical software Minitab version 14.0 (*p < 0.05 and **p < 0.01). All the experiments performed thrice and represented as SD.

## 3 RESULTS AND DISCUSSION

### 3.1 Growth of *Aspergillus niveus*

**FIGURE 1:**
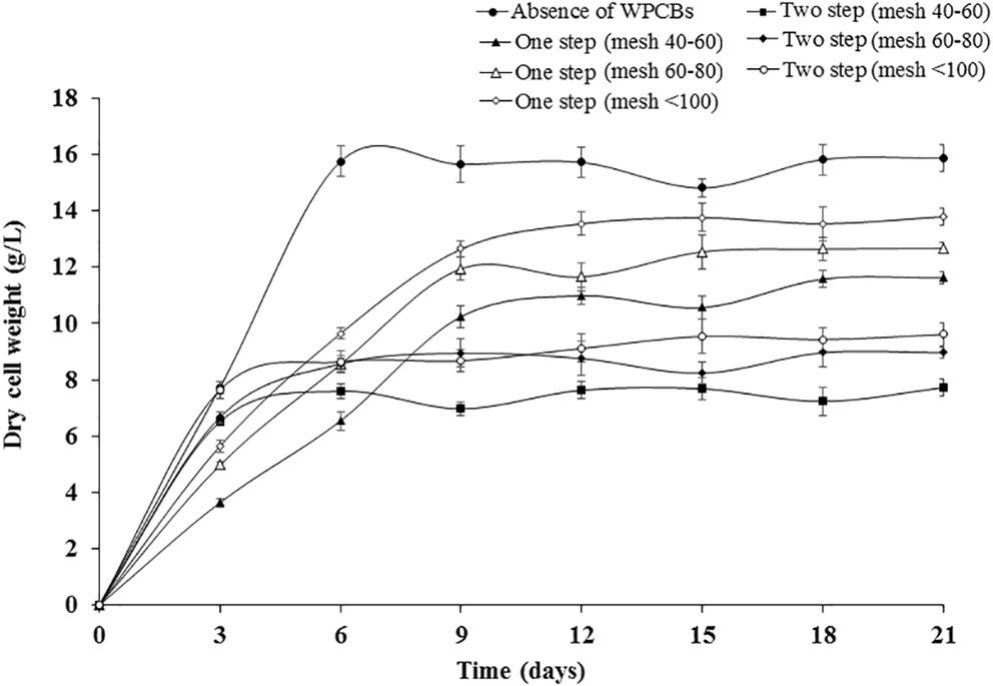
Dry cell weight of *Aspergillus niveus* in the absence and the presence of WPCBs particles at 37°C and 180 rpm

*Aspergillus* species are well‐known for the production of organic acids [31]. Figure 1 represents the dry cell weight of *A. niveus* in the presence and absence of 0.1% (wt/vol) of WPCBs of different particle size. The variation in dry cell weight of *A. niveus*, with respect to various conditions, was found to be statistically significant. In the presence of WPCBs, the growth of *A. niveus* was found to be less when compared with the growth of *A. niveus* in the absence of WPCBs. Initially, the growth of *A. niveus* was found to be slow for 3 days, but a further increase in the dry cell weight was observed and that finally reached a maximum of 15.82 ± 0.54 g/L in 6 days in the absence of WPCBs. Furthermore, the dry cell weight was found to be about constant up to 21 days.

When particle sizes of 40–60 mesh were used in one‐step bioleaching, growth of *A. niveus* was found to be slow for the first 9 days and then steadily increased and reached a maximum of 10.85 ± 0.45 g/L in 12 days. Furthermore, the dry cell weight was found to be about constant up to 21 days. When other particle sizes (60–80 and <100 mesh) were used, trends of *A. niveus* growth were similar to those of 40–60 mesh size particles used. But the lowest dry cell weight was observed when the particle sizes of 40–60 mesh were used.

In the two‐step bioleaching method, when particle sizes of 40–60 mesh were used, growth of *A. niveus* was slow for 3 days and then steadily increased and reached 7.63 ± 0.35 g/L in 12 days. Furthermore, the dry cell weight was found to be constant up to 21 days. Similar trends were noticed for other two‐size range of particles. Here, lowest dry cell weight was observed, when the particle size of 40–60 mesh was used. The growth of *A. niveus* was hindered by heavy metals present in the WPCBs. However, heavy metals have toxic effects on microbial growth [32].

**FIGURE 2:**
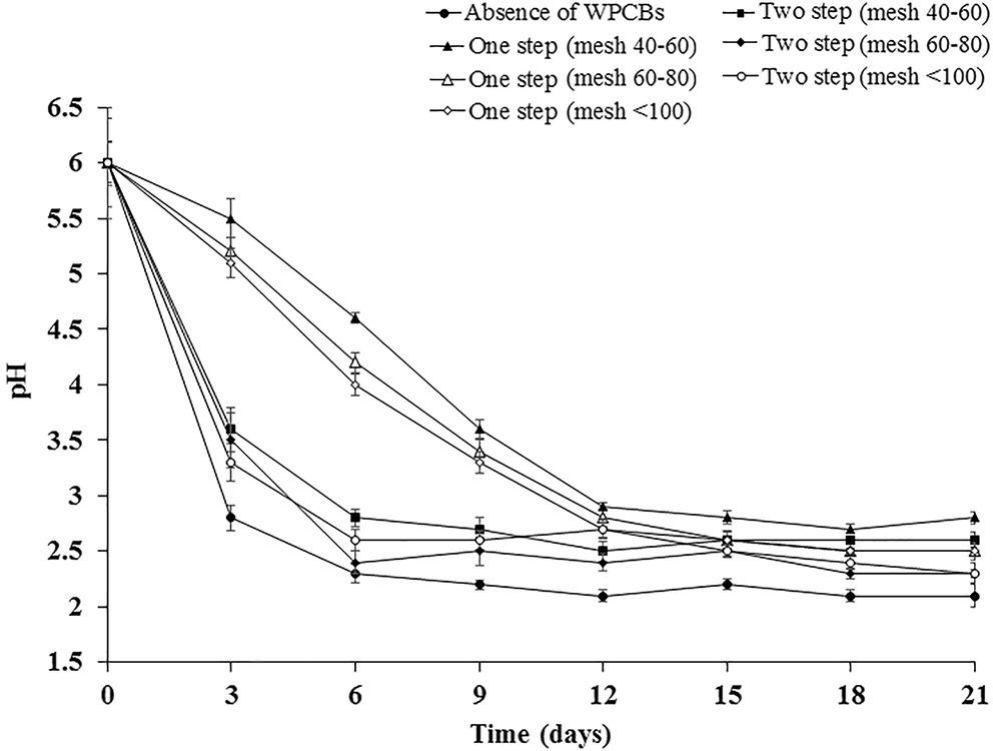
Medium pH during the growth of *Aspergillus niveus* in the absence and presence of WPCBs particles at 37°C and 180 rpm

The changes in pH throughout the fungal growth were recorded as shown in Figure 2. Variation in pH during the growth of *A. niveus* with respect to various conditions was found to be statistically significant. It was observed that reduction in pH occurred from 6.0 to 2.3 for 6 days, in the absence of WPCBs. After 6 days, the pH remained constant at 2.2 approximately. Likewise, during the two‐step bioleaching, reduction in pH occurred from 6.0 to 2.8 for 6 days, and when particles from 40–60 mesh were used beyond 6 days, the pH remained constant. When other particle sizes (60–80 and <100 mesh) were used, the trend of pH was similar to those where 40–60 mesh size particles were used. However, during one‐step leaching, a gradual decrease in pH was observed from 6.0 to 2.9 for 12 days and the pH remained constant around 2.7 beyond 21 days when the particles from 40–60 mesh were used. The reduction in pH is due to the acidification that occurr due to the organic acids produced [32]. When other particle sizes (60–80 and <100 mesh) were used, the trend of pH was similar to case where 40–60 mesh size particles used.

### 3.2 Production of organic acids by *Aspergillus niveus*

**FIGURE 3:**
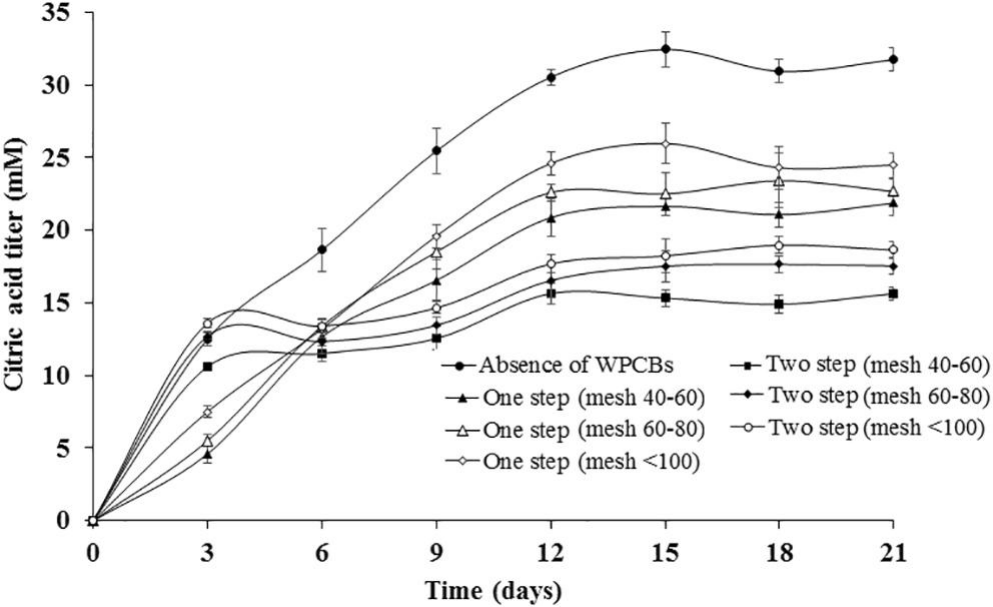
Amount of citric acid produced during *Aspergillus niveus* growth in the absence and presence of WPCBs particles at 37°C and 180 rpm

Figure 3 represents the amount of citric acid produced by *A. niveus* in the presence and absence of WPCBs. The variation in the amount of citric acid produced by *A. niveus* with respect to various conditions was found to be statistically significant using Student's *t*‐test. The amount of citric acid generated by *A. niveus* increased and reached 30.54 ± 1.44 mM in 12 days in absence of WPCBs. After 12 days, the amount of citric acid was nearly constant about 31.55 ± 2.56 mM and remained similar up to 21 days.

When particle sizes of 40–60 mesh were used in one‐step bioleaching approach, citric acid reached a maximum concentration level of 20.87 ± 1.38 mM in 12 days and beyond that, the amount of citric acid was relatively constant up to 21 days. Analogous movements were observed for other particle size. The amount of citric acid produced during one‐step leaching was lower in the absence of WPCBs. When particles of size 40–60 mesh were used in the two‐step leaching, the amount of citric acid concentration reached 15.64 ± 0.75 mM after 12 days which was less than the one‐step bioleaching. Notably, the amount of citric‐acid produced during the initial stage of the two‐step leaching, before the introduction of the WPCBs powder of, was about 10.65 ± 0.45 mM in 3 days, which was of a comparatively similar value obtained without the presence of WPCBs. Thus, the presence of WPCBs delayed the secretion of citric acid.

**FIGURE 4:**
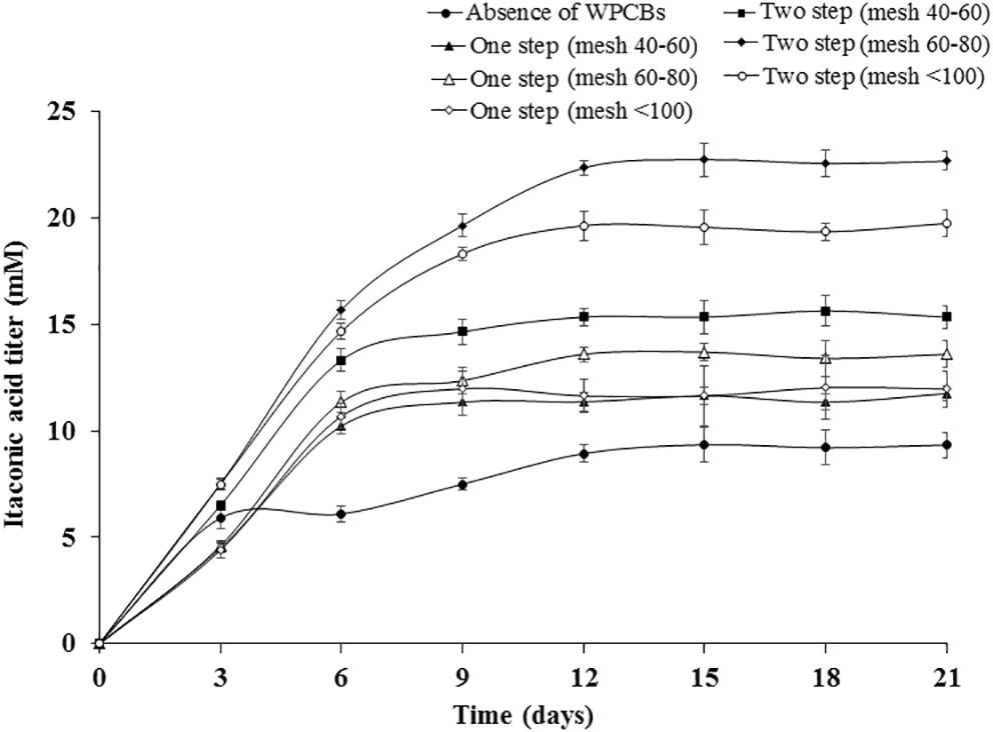
Amount of itaconic acid produced during *Aspergillus niveus* growth in the absence and presence of WPCBs particles at 37°C and 180 rpm

Itaconic acid produced by *A. niveus* was increased to 8.94 ± 0.47 mM after 12 days in the absence of WPCBs as shown in Figure 4. Variation in the amount of itaconic acid produced with respect to various circumstances was found to be statistically significant using Student's *t*‐test in Figure 4. After 12 days, the amount of itaconic acid was nearly constant about 9.22 ± 0.57 mM and remained so up to 21 days. When particle sizes of 40–60 mesh were used in the one‐step bioleaching approach, itaconic acid reached the maximum concentration of 11.35 ± 0.64 mM after 9 days and beyond that, the amount of itaconic acid was relatively constant up to 21 days. The amount of itaconic acid produced during the one‐step leaching was higher in the absence of WPCBs.

**FIGURE 5:**
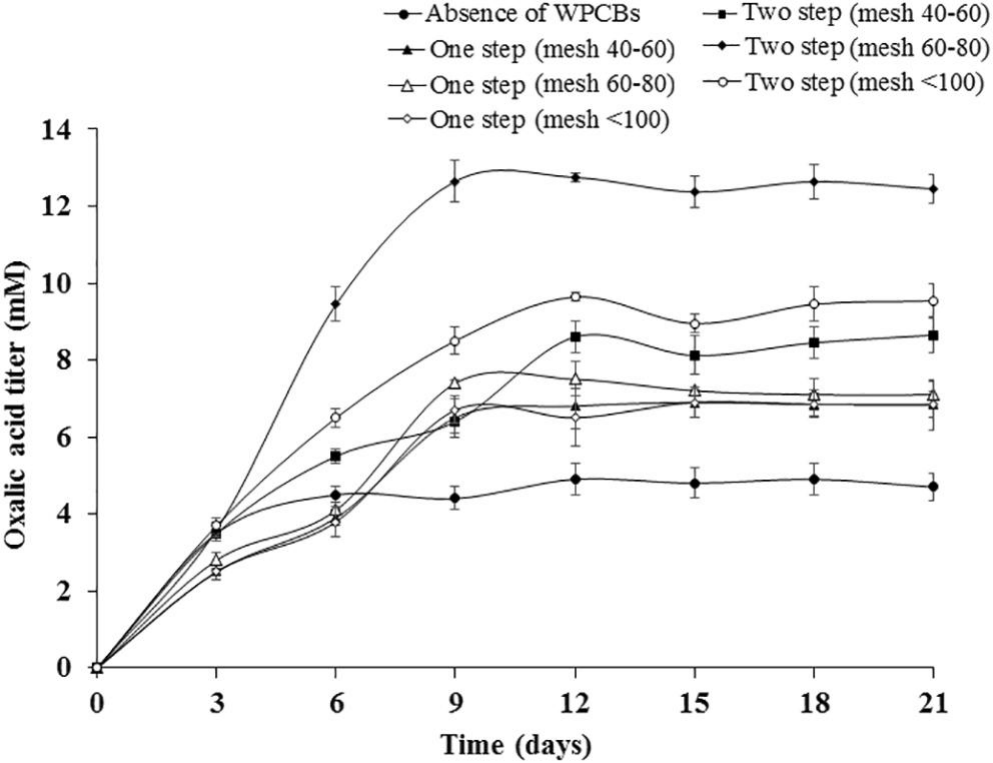
The amount of oxalic acid produced during *Aspergillus niveus* growth in the absence and the presence of WPCBs particles at 37°C and 180 rpm

In the two‐step leaching, after 12 days, itaconic acid concentration reached a maximum of 22.35 ± 0.87 mM when particles of 60–80 mesh were used which was higher than the one‐step bioleaching. Thus, presence of WPCBs prompted the secretion of itaconic acid. Similarly, oxalic acid secreted by *A. niveus* was increased in the presence of WPCBs when compared in the absence of WPCBs (Figure 5). The variation in the amount of oxalic acid produced by *A. niveus* under various circumstances was found to be statistically significant using Student's *t*‐test in Figure 5. Various parameters like initial pH, inoculum concentration, carbon source, nitrogen source, presence of trace metals and heavy metals might affect the production of organic acids [33, 34, 35]. When *A. niveus* was exposed to the toxicity of WPCBs powder, the secretion of itaconic acid and oxalic acid changed. It might be due to the developed performance of *A. niveus* to enhance the toxicity tolerance and it could be achieved through the secretion of metabolites. Prominently, study related to the statistical optimization of organic acids produced by *A. niveus* need to be investigated in future.

### 3.3 Bioleaching of valuable metals

The organic acids produced by *A. niveus* can contribute to the bioleaching of valuable metals. Initially, the protons are released from the organic acids on dissociation and the released protons attach with metal ions [36] and further result in the formation of the complex. Metal ions are removed from the surface by the role of the produced organic acids [37] and biochar [38]. As expected, the organic acids produced by *A. niveus* bioleached Zn, Cu and Ni from various particle sizes of WPCBs.

**FIGURE 6:**
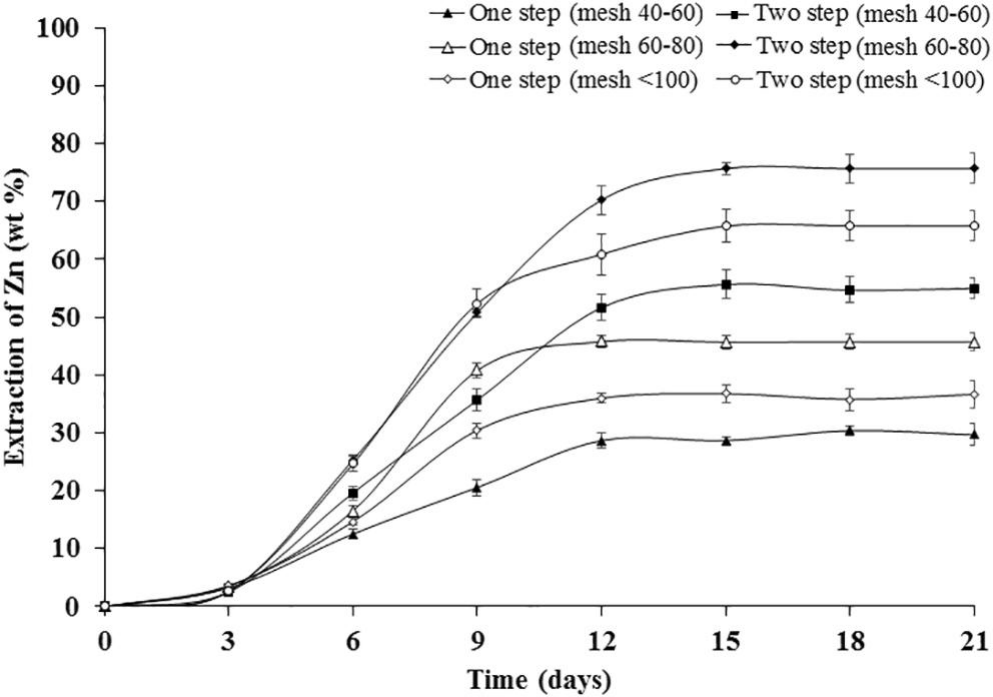
Bioleaching of zinc from WPCBs using *Aspergillus niveus* at 37°C and 180 rpm

Figure 6 represents bioleaching of Zn from WPCBs of various particle sizes using one‐step and two‐step bioleaching approaches. Variations in the extraction of Zn from the WPCBs using different bioleaching approaches were found to be statistically significant using Student's *t*‐test as in Figure 6. The extraction of Zn increased in 12 days and remained consistent for 21 days in all approaches of bioleaching. The two‐step leaching approach with particles of 60–80 mesh resulted in the highest Zn of Zn 75.66 ± 2.64 wt% in 21 days, showing the maximum extraction of Zn, whereas the one‐step approach with particles of 40–60 mesh size resulted in the lowest extraction of Zn (29.67 ± 1.54 wt %) in 21 days. It was consistent with the result of the dry cell weight of *A. niveus* as shown in Figure 1. Among the one‐step leaching approaches, particles of WPCBs with 60–80 size showed maximum extraction of Zn and minimum extraction of Zn with particles of 40–60 mesh size. It might be due to the surface area of the WPCBs particles which could attribute to the efficiency of leaching [39]. Prominently, toxicity due to metal ions might be exerted by particles of different sizes [40].

**FIGURE 7:**
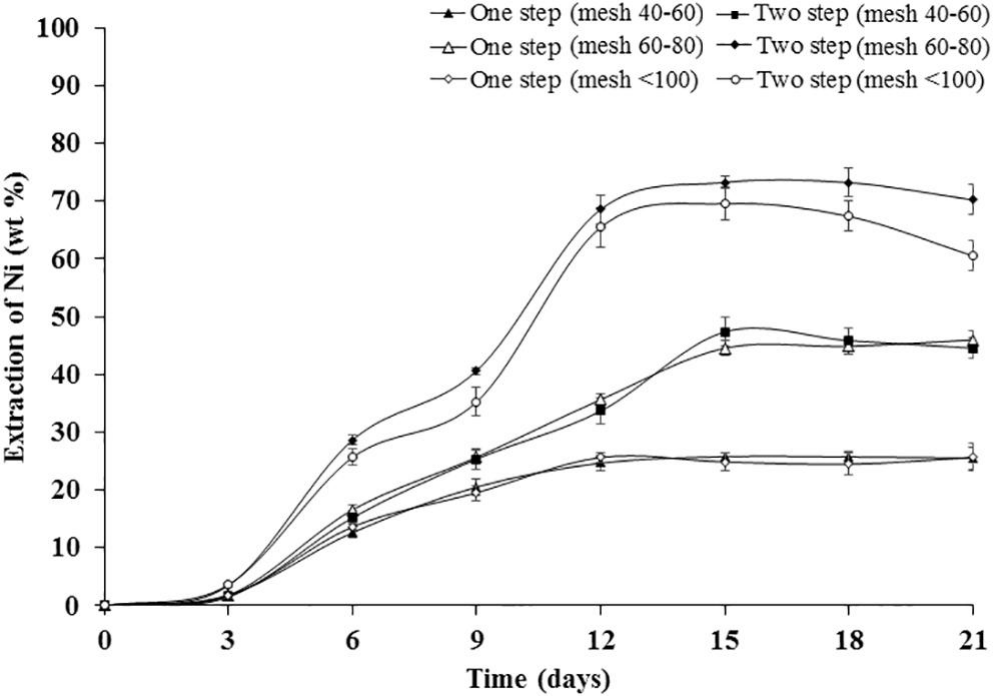
Bioleaching of nickel from WPCBs using *Aspergillus niveus* at 37°C and 180 rpm

Figure 7 represents bioleaching of Ni from WPCBs of different particle dimensions using one‐step and two‐step bioleaching approaches. The variation in the extraction of Ni from the WPCBs by different bioleaching approaches was found to be statistically significant using Student's *t*‐test in Figure 7. During the two‐step leaching approach with particles of 60–80 and <100, removal of Ni was found to increase from 9  to 18 days and further decrease after 18 days. The decrease in Ni extraction may be accounted due to the formation of nickel oxalate [41]. The maximum nickel extraction was observed to be 73.58 ± 1.75 wt% in 15 days in the two‐step leaching with the particles size of 60–80 mesh. When compared with one‐step leaching, maximum extraction of Ni was found using two‐step leaching. The lowest nickel extraction was observed in one‐step leaching with particles of 40–60. The trend obtained in one‐step leaching with particles of less than 100 mesh was similar to that of one‐step leaching with particle sizes of 40–60 mesh. It was also proved that during one‐step leaching particles of 40–60 and <100, the extraction of Ni was more or less similar.

**FIGURE 8:**
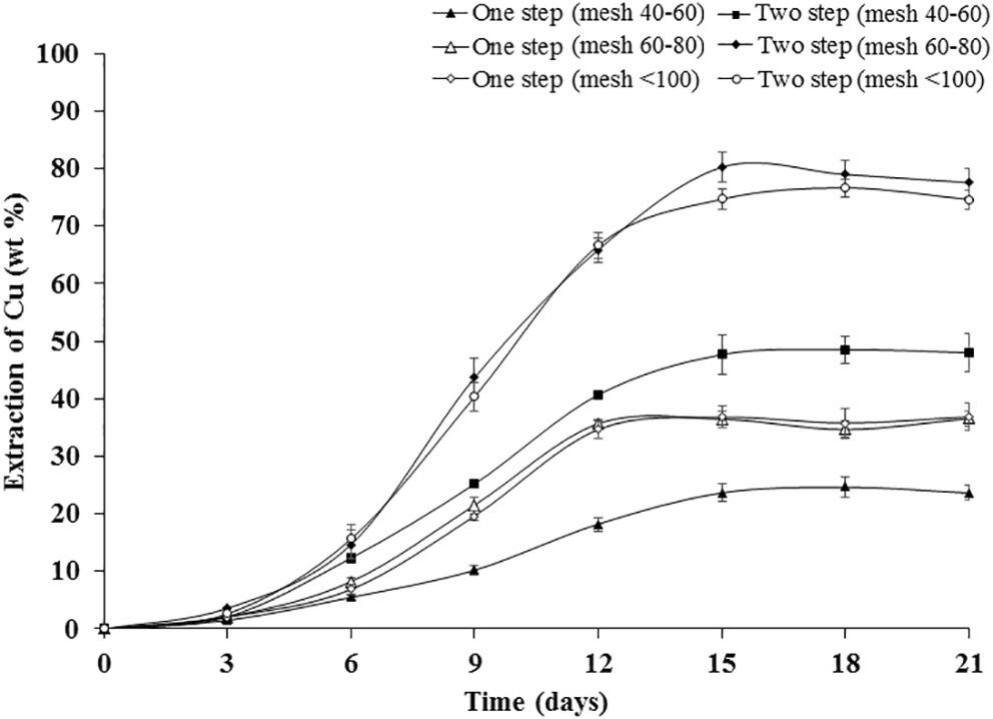
Bioleaching of copper from WPCBs using *Aspergillus niveus* at 37°C and 180 rpm

Figure 8 represents bioleaching of Cu from the WPCBs of different particle dimensions using one‐step and two‐step bioleaching approaches. The variation in the recovery of Cu from WPCBs using different bioleaching approaches was found to be statistically significant. The removal of Cu increased in 12 days and it was nearly constant up to 21 days in all approaches of bioleaching. The highest copper extraction was found to be 80.25 ± 2.54 wt% in 15 days in two‐step leaching with particle sizes of mesh being 60–80. Ghose and Paul [42] reported that *Aspergillus humicola* species was employed for bioleaching of nickel. Oxalic acid was able to produce oxalates which affect the bioleaching of valuable metals [7]. Other organic acids produced by *Aspergillus* species can leach metal ions from the WPCBs [43]. *A. niveus* used in this study was reported to produce itaconic acid from glycerol and the algal biomass hydrolysate.

**FIGURE 9:**
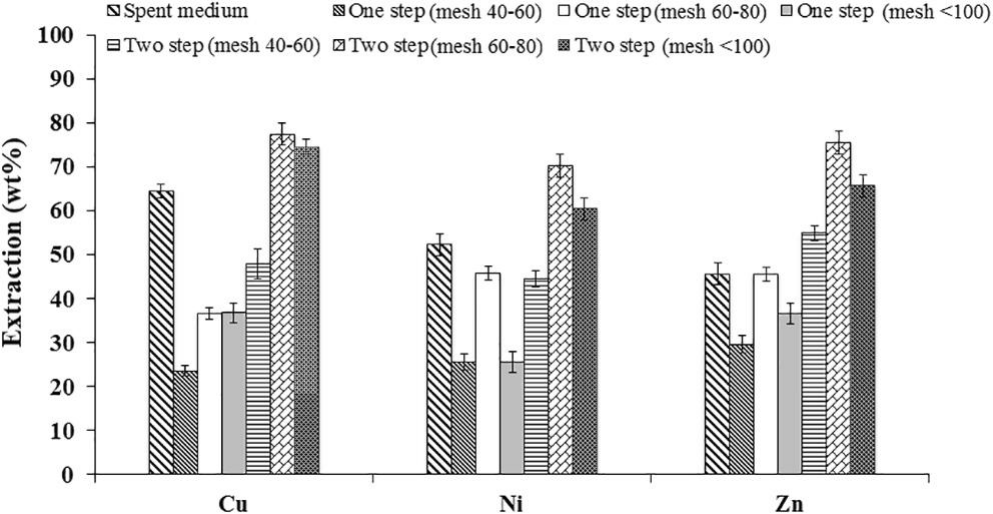
Bioleaching of metals from WPCBs using *Aspergillus niveus*

The comparison of all the bioleaching approaches is represented in Figure 9. The variation in metal extraction from WPCBs by different bioleaching approaches was found to be statistically significant using Student's *t*‐test as in Figure 9. It was found that the bioleached amount of all the three metal ions was more than 20% in all the approaches of bioleaching. Maximum recovery of Ni and Zn were found to be 70.43 ± 2.64 wt% and 75.69 ± 2.8 wt%, respectively. Furthermore, maximum retrieval of Cu was found to be 77.25 ± 2.4 wt% during the two‐step leaching of 60–80 mesh particles.

### 3.4 Chemical leaching

**FIGURE 10:**
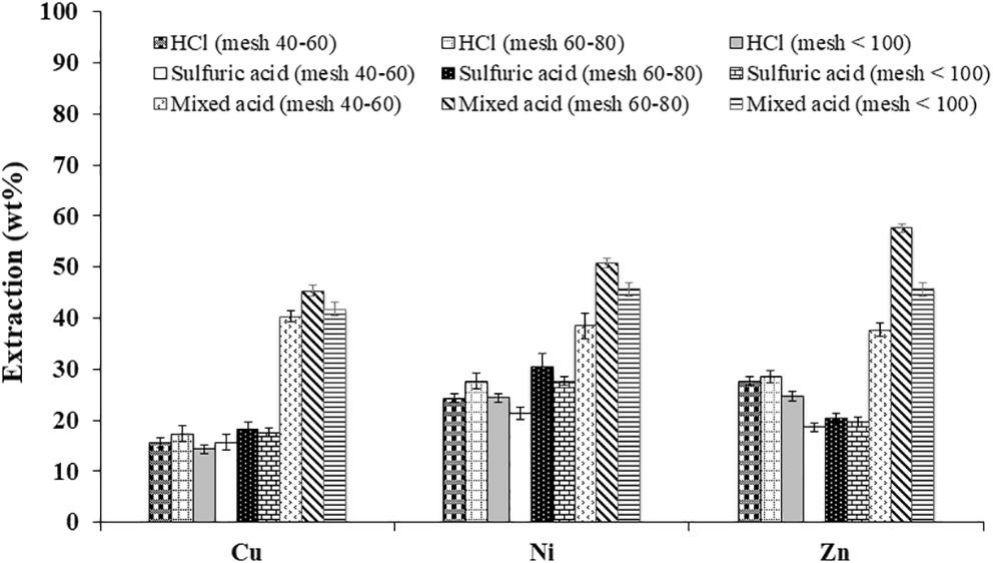
Chemical leaching of metals from WPCBs

Different particle sizes of WPCBs were chemically leached using strong acids and analytical grade organic acid mixture containing mixed organic acids consisting of 50 mM citric acid, 30 mM itaconic acid and 20 mM oxalic acid. The proportions were selected based on the trial and error method. Among the two inorganic acids, sulphuric acid showed the maximum leaching of Cu when compared with hydrochloric acid, when the particles were of size 60–80 as shown in Figure 10. A similar pattern was observed for the leaching of Ni. Leaching of Zn in hydrochloric acid was increased in sulphuric acid. The variations in the extraction of metals from the WPCBs using different chemical leaching approaches were found to be statistically significant using Student's *t*‐test in Figure 10. When different particles of WPCBs were exposed to mixture containing 50 mM citric acid, 30 mM itaconic acid and 20 mM oxalic acid, the maximum recovery of all three metals occurred at particles of mesh size 60–80. As a comparison, usage of organic acids was found to increase leaching of valuable metals when compared with 100 mM of inorganic acids. This established the efficacy of organic acids.

### 3.5 Comparison between bioleaching and chemical leaching

Leaching of Cu, Ni and Zn using organic acids produced by *A. niveus* and chemical leaching are represented in Figures 9 and 10. It is obvious that generation of different particles size of WPCBs had a significant result on the recovery of valuable metals. Highest recovery of valuable metals was obtained when particles in the size of mesh 60–80 were used. Organic acids produced by *A. niveus* showed a greater efficiency on leaching of Cu, Ni and Zn. Moreover, the contribution of individual organic acids produced by *A. niveus* can be performed. Furthermore, the optimization of valuable metal leaching using organic acids produced by *A. niveus* can also be explored in future.

### 3.6 Surface morphology of waste circuit boards

**FIGURE 11:**
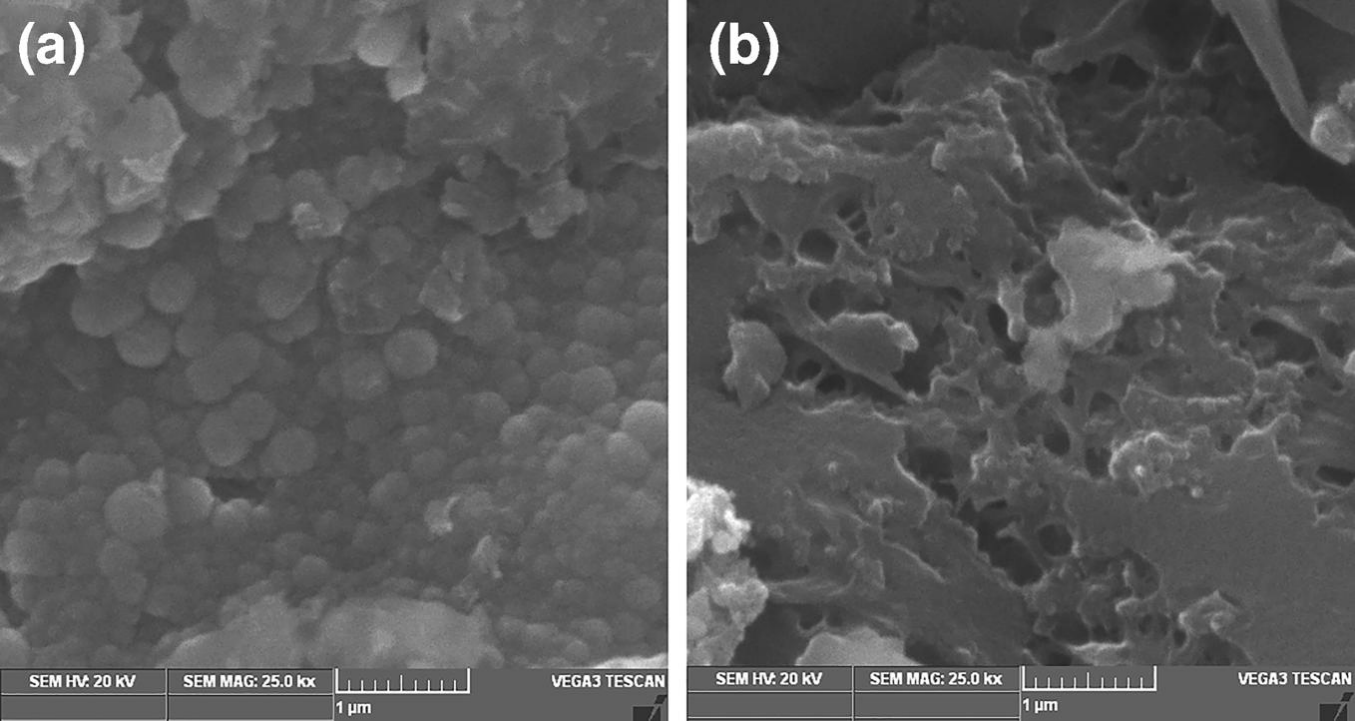
SEM images of WPCB power (a) before and (b) after bioleaching

To examine the outcome of bioleaching on the surface morphology of WPCBs, SEM analysis was performed. Figure 11 represents the surface morphology of WPCBs particle of size 40–60 in a two‐step approach before bioleaching, which had a smooth surface. Bioleaching using 40–60 mesh particle sizes of WPCBs resulted in the alteration of surface morphology with remarkable changes as shown in Figure 11. Thus, *A. niveus* that produced organic acids eroded metal contents to the solution phase. Similarly, Horeh et al. [43] reported that *Aspergillus niger* was used for bioleaching of metals from waste batteries made of lithium‐ion based. Wang et al. [28] disclosed that the particle size had a significant role in the efficiency of bioleaching of the metal.

### 3.7 Cost analysis

Nowadays, recycling of WPCBs is a very complicated process due to various components. Among the various e‐waste, generation of WPCBs is estimated to about 6% which in turn comes to 0.5 million tons annually [23, 46]. Importantly, WPCBs contain precious metals like Zn, Cu and Ni, which are of greater value. The scope of researches on bioleaching of valuable metals from WPCBs or other electronic waste is tremendous. Moreover, bioleaching method is learnt to be a cost‐effective method when compared with other conventional methods of leaching of valuable metals [46]. The cost‐effective substrate can be used for the production of organic acids using fungal species. The main drawback in the bioleaching of metals from WPCBs is the production of toxic elements [47]. Thus, this study, the generation of different particle sizes of WPCBs, proved to bioleach Cu, Zn and Ni from WPCBs by using organic acids produced by *A. niveus*.

## 4 CONCLUSION

In this study, *A. niveus* was employed for the recovery of valuable metals from pre‐treated WPCBs. Sucrose was utilised as the substrate for organic acid production using *A. niveus*. The effect of various particle sizes on bioleaching of valuables like Zn, Cu and Ni was studied. Variations in dry cell weight and organic acid productions were observed in the presence of WPCBs with different particle sizes. The increase in itaconic acid and oxalic acid produced by *A. niveus* reached a maximum titre of 22.35 ± 0.87 mM for itacoinc acid and 12.75 ± 0.54 mM for oxalic acid in 12 days, when particle sizes of 60–80 mesh were used during the two‐step bioleaching. The decrease in citric acid titer was found both in the one‐step and the two‐step bioleaching approaches. Minimum citric acid titre was found to be 15.64 ± 0.45 mM with particle sizes of 40–60 mesh in the two‐step bioleaching. Maximum recoveries of 75.66% Zn, 73.58% Ni and 80.25% Cu from WPCBs were achieved in 15 days in the two‐step leaching using particles of mesh size 60–80. Different methods like immobilisation, optimal treatment and biofilm formation require attention to improve the bioleaching efficiency.

## References

[1] Ikhlayel, M. : An integrated approach to establish e‐waste management systems for developing countries. J. Clean. Prod. 170, 119–130 (2018)

[2] Yu, Z. , Han, H. , Feng, P. , et al.: Recent advances in the recovery of metals from waste through biological processes. Bioresour. Technol. 297, 122416 (2020) 31786035 10.1016/j.biortech.2019.122416

[3] Santo, E. , et al.: Quantification of E‐waste : a case study in Federal. Int. J Env. Ecol Eng. 11(2), 195–203 (2017)

[4] Arshadi, M. , Mousavi, S.M. : Simultaneous recovery of Ni and Cu from computer‐printed circuit boards using bioleaching: statistical evaluation and optimization’Bioresour. Technol. 174, 233–242 (2014) 25463804 10.1016/j.biortech.2014.09.140

[5] Jagannath, A. , Vidya Shetty, K. , Saidutta, M.B. : Bioleaching of copper from electronic waste using *Acinetobacter* sp. Cr B2 in a pulsed plate column operated in batch and sequential batch mode. J. Environ. Chem. Eng. 5(2), 1599–1607 (2017)

[6] Hadi, P. , Xu, M. , Lin, C.S.K. , Hui, C.W. , McKay, G. : Waste printed circuit board recycling techniques and product utilization. J. Hazard. Mater. 283, 234–243 (2015) 25285997 10.1016/j.jhazmat.2014.09.032

[7] Cui, H. , Anderson, C.G. : Literature review of hydrometallurgical recycling of printed circuit boards (PCBs). J. Adv Chem Eng. 6(1), 1–11 (2016)

[8] Kaliyaraj, D. , Rajendran, M. , Angamuthu, V. , et al.: Bioleaching of heavy metals from printed circuit board (PCB) by Streptomyces albidoflavus TN10 isolated from insect nest. Bioresour. Bioprocess. 6(1), 47 (2019)

[9] Işıldar, A. , van de Vossenberg, J. , Rene, E.R. , van Hullebusch, E.D. , Lens, P.N.L. : Two‐step bioleaching of copper and gold from discarded printed circuit boards (PCB). Waste Manag. 57, 149–157 (2016) 26704063 10.1016/j.wasman.2015.11.033

[10] Awasthi, A.K. , Zeng, X. , Li, J. : Integrated bioleaching of copper metal from waste printed circuit board–a comprehensive review of approaches and challenges. Environ. Sci. Pollut. Res. 23(21), 21141–21156 (2016) 10.1007/s11356-016-7529-9 27678000

[11] Guo, Q. , Yue, X. , Wang, M. , Liu, Y. : Pyrolysis of scrap printed circuit board plastic particles in a fluidized bed. Powder Technol. 198(3), 422–428 (2010)

[12] Havlik, T. , et al.: Leaching of copper and tin from used printed circuit boards after thermal treatment. J. Hazard. Mater. 183(1–3), 866–873 (2010) 20800354 10.1016/j.jhazmat.2010.07.107

[13] Cui, J. , Zhang, L. : Metallurgical recovery of metals from electronic waste: a review. J. Hazard. Mater. 158(2–3), 228–256 (2008) 18359555 10.1016/j.jhazmat.2008.02.001

[14] Jadhav, U. , Su, C. , Hocheng, H. : Leaching of metals from large pieces of printed circuit boards using citric acid and hydrogen peroxide. Environ. Sci. Pollut. Res 23(23), 24384–24392 (2016) 10.1007/s11356-016-7695-9 27655620

[15] Wang, J. , Xu, Z. : Disposing and recycling waste printed circuit boards: disconnecting, resource recovery, and pollution control. Environ. Sci Technol. 49(2), 721–733 (2015) 25525865 10.1021/es504833y

[16] Wang, F. , et al.: An: A two‐step leaching method designed based on chemical fraction distribution of the heavy metals for selective leaching of Cd, Zn, Cu, and Pb from metallurgical sludge. Environ. Sci. Pollut. Res. 25(2), 1752–1765 (2018) 10.1007/s11356-017-0471-7 29101700

[17] Aung, K.M.M. , Ting, Y.P. : Bioleaching of spent fluid catalytic cracking catalyst using *Aspergillus niger* . J. Biotechnol. 116(2), 159–170 (2005) 15664080 10.1016/j.jbiotec.2004.10.008

[18] Lo, Y.C. , Cheng, C.L. , Han, Y.L. , Chen, B.Y. , Chang, J.S. : Recovery of high‐value metals from geothermal sites by biosorption and bioaccumulation. Bioresour. Technol. 160, 182–190 (2014) 24581863 10.1016/j.biortech.2014.02.008

[19] Latorre, M. , Cortés, M.P. , Travisany, D. , et al.: The bioleaching potential of a bacterial consortium. Bioresour. Technol. 218, 659–666 (2016) 27416516 10.1016/j.biortech.2016.07.012

[20] Brombacher, C. , Bachofen, R. , Brandl, H. : Biohydrometallurgical processing of solids: a patent review. Appl. Microbiol. Biotechnol 48(5), 577–587 (1997)

[21] Gu, W. , Bai, J. , Lu, L. , et al.: Improved bioleaching efficiency of metals from waste printed circuit boards by mechanical activation. Waste Manag. 98, 21–28 (2019) 31421486 10.1016/j.wasman.2019.08.013

[22] Li, J. , et al.: Bioleaching of gold from waste printed circuit boards by alkali‐tolerant *Pseudomonas* fluorescens. Hydrometallurgy. 194(3), 529–539 (2020)

[23] Arshadi, M. , Mousavi, S.M. : Multi‐objective optimization of heavy metals bioleaching from discarded mobile phone PCBs: simultaneous Cu and Ni recovery using *Acidithiobacillus ferrooxidans* . Sep. Purif. Technol. 147, 210–219 (2015)

[24] Priya, A. , Hait, S. : Extraction of metals from high grade waste printed circuit board by conventional and hybrid bioleaching using *Acidithiobacillus ferrooxidan* . Hydrometallurgy. 177, 132–139 (2018)

[25] Yang, J. , Wang, Q. , Wang, Q. , Wu, T. : Heavy metals extraction from municipal solid waste incineration fly ash using adapted metal tolerant *Aspergillus niger* . Bioresour. Technol. 100(1), 254–260 (2009) 18599287 10.1016/j.biortech.2008.05.026

[26] Qu, Y. , Lian, B. , Mo, B. , Liu, C. : Bioleaching of heavy metals from red mud using *Aspergillus niger* . Hydrometallurgy. 136, 71–77 (2013)

[27] Bahaloo‐Horeh, N. , Mousavi, S.M. : Enhanced recovery of valuable metals from spent lithium‐ion batteries through optimization of organic acids produced by *Aspergillus niger* . Waste Manag. 60, 666–679 (2017) 27825532 10.1016/j.wasman.2016.10.034

[28] Gnanasekaran, R. , Dhandapani, B. , Iyyappan, J. : Improved itaconic acid production by *Aspergillus niveus* using blended algal biomass hydrolysate and glycerol as substrates. Bioresour. Technol. 283, 297–302 (2019) 30921582 10.1016/j.biortech.2019.03.107

[29] Faraji, F. , Golmohammadzadeh, R. , Rashchi, F. , Alimardani, N. : Fungal bioleaching of WPCBs using *Aspergillus niger*: observation, optimization and kinetics. J. Environ. Manage. 217, 775–787 (2018) 29660703 10.1016/j.jenvman.2018.04.043

[30] Kim, M.J. , Seo, J.Y. , Choi, Y.S. , Kim, G.H. : Bioleaching of spent Zn‐Mn or Ni‐Cd batteries by *Aspergillus* species. Waste Manag., 51, 168–173 (2016) 26584557 10.1016/j.wasman.2015.11.001

[31] Yang, L. , Lübeck, M. , Lübeck, P.S. : *Aspergillus* as a versatile cell factory for organic acid production. Fungal. Biol Rev. 31(1), 33–49 (2017)

[32] Burgstaller, W. , Schinner, F. : Leaching of metals with fungi. J. Biotechnol. 27(2), 91–116 (1993)

[33] Wang, Y. , et al.: Effects of nitrogen availability on polymalic acid biosynthesis in the yeast‐like fungus *Aureobasidium pullulans* . Microb. Cell Fact. 15(1), 146 (2016) 27549441 10.1186/s12934-016-0547-y PMC4994417

[34] Ozdal, M. , Kurbanoglu, E.B. : Citric acid production by Aspergillus niger from Agro‐industrial by‐products: molasses and chicken feather peptone’ Waste Biomass Valoriz. 10(3), 631–640 (2019)

[35] Dhandapani, B. , Vishnu, D. , Murshid, S. , et al.: Production of lactic acid from industrial waste paper sludge using *Rhizopus oryzae* MTCC5384 by simultaneous saccharification and fermentation. Chem. Eng. Commun. pp. 1–9 (2019)

[36] Golmohammadzadeh, R. , Rashchi, F. , Vahidi, E. : Recovery of lithium and cobalt from spent lithium‐ion batteries using organic acids: process optimization and kinetic aspects. Waste Manag., 64, 244–254 (2017) 28365275 10.1016/j.wasman.2017.03.037

[37] Amiri, F. , Mousavi, S.M. , Yaghmaei, S. , Barati, M. : Bioleaching kinetics of a spent refinery catalyst using *Aspergillus niger* at optimal conditions. Biochem. Eng J. 67, 208–217 (2012)

[38] Wang, S. , et al.: Enhanced bioleaching efficiency of metals from E‐wastes driven by biochar. J. Hazard. Mater. 320, 393–400 (2016) 27585271 10.1016/j.jhazmat.2016.08.054

[39] Nemati, M. , Lowenadler, J. , Harrison, S.T.L. : Particle size effects in bioleaching of pyrite by acidophilic thermophile *Sulfolobus metallicus* (BC). Appl. Microbiol. Biotechnol. 53(2), 173–179 (2000) 10709979 10.1007/s002530050005

[40] Zhu, N. , Xiang, Y. , Zhang, T. , et al.: Bioleaching of metal concentrates of waste printed circuit boards by mixed culture of acidophilic bacteria. J. Hazard. Mater. 192(2), 614–619 (2011) 21683521 10.1016/j.jhazmat.2011.05.062

[41] Magyarosy, A. , et al.: Nickel accumulation and nickel oxalate precipitation by *Aspergillus niger* . Appl. Microbiol. Biotechnol. 59(2–3), 382–388 (2002) 12111174 10.1007/s00253-002-1020-x

[42] Ghosh, S. , Paul, A.K. : Bioleaching of nickel by *Aspergillus humicola* SKP102 isolated from Indian lateritic overburden. J. Sustain. Min. 15(3), 108–114 (2016)

[43] Horeh, N.B. , Mousavi, S.M. , Shojaosadati, S.A. : Bioleaching of valuable metals from spent lithium‐ion mobile phone batteries using *Aspergillus Niger* . J. Power Sources. 320, 257–266 (2016)

[44] Wang, X. , Sun, Z. , Liu, Y. , et al.: Effect of particle size on uranium bioleaching in column reactors from a low‐grade uranium ore’Bioresour. Technol. 281, 66–71 (2019) 30798088 10.1016/j.biortech.2019.02.065

[45] Gu, W. , Bai, J. , Dong, B. , et al.: Catalytic effect of graphene in bioleaching copper from waste printed circuit boards by *Acidithiobacillus ferrooxidans* . Hydrometallurgy. 171, 172–178 (2017)

[46] Baniasadi, M. , Vakilchap, F. , Bahaloo‐Horeh, N. , Mousavi, S.M. , Farnaud, S. : Advances in bioleaching as a sustainable method for metal recovery from e‐waste: a review’J. Ind. Eng. Chem. 76, 75–90 (2019)

[47] Bryan, C.G. , Watkin, E.L. , McCredden, T.J. , Wong, Z.R. , Harrison, S.T.L. , Kaksonen, A.H. : The use of pyrite as a source of lixiviant in the bioleaching of electronic waste. Hydrometallurgy. 152, 33–43 (2015)

